# Substantial discrepancies in dengue case estimates between the Global Burden of Disease Study and Taiwan Centers for Disease Control

**DOI:** 10.1093/jtm/taae009

**Published:** 2024-01-17

**Authors:** Sin Yee Lee, Hsin-I Shih, Chwan-Chuen King, Tsung-Hsueh Lu, Yu-Wen Chien

**Affiliations:** Department of Public Health, College of Medicine, National Cheng Kung University, Tainan 701, Taiwan; Department of Emergency Medicine, National Cheng Kung University Hospital, College of Medicine, National Cheng Kung University, Tainan 704, Taiwan; School of Medicine, College of Medicine, National Cheng Kung University, Tainan 701, Taiwan; Institute of Epidemiology and Preventive Medicine, College of Public Health, National Taiwan University, Taipei City 100, Taiwan; Department of Public Health, College of Medicine, National Cheng Kung University, Tainan 701, Taiwan; Department of Public Health, College of Medicine, National Cheng Kung University, Tainan 701, Taiwan; Department of Occupational and Environmental Medicine, National Cheng Kung University Hospital, College of Medicine, National Cheng Kung University, Tainan 704, Taiwan

## Abstract

Taiwan’s dengue cases vary annually, peaking in infrequent epidemics, which differ substantially from the Global Burden of Disease Study’s (GBD’s) projections. Although the GBD study provides invaluable insights into global health trends, its modelling approach fails to capture the dynamic change of dengue transmission.

The Global Burden of Disease Study (GBD), launched in 1991, is an ongoing effort to quantify mortality, morbidity and disability arising from major diseases, injuries and risk factors.^1^^,^^2^ GBD estimates, based on modelling because of scarce reliable data, have become crucial for research and policy-making. However, despite advancements in modelling techniques, their credibility is limited by inconsistent availability and quality of underlying data.^3^

Dengue poses a significant public health challenge. Yang *et al.*^4^, using GBD 2019 data, reported that Taiwan experienced the second-fastest rise in dengue-related age-standardized death rate from 1990 to 2019, with an estimated annual percentage change of 24.47. This report warrants closer examination, as the Taiwan Centers for Disease Control (Taiwan CDC) recorded only 228 deaths during the 2015 outbreak, the most severe dengue epidemic in recent decades.^5^ Therefore, this study aimed to compare the number of laboratory-confirmed dengue cases recorded by Taiwan CDC with the GBD estimates for Taiwan.

In Taiwan, physicians are mandated to report patients with symptoms suspected to be dengue infection to the CDC and take blood samples for laboratory confirmation. The laboratory confirmation criteria include viral isolation, positive real-time reverse transcription-polymerase chain reaction, 4-fold increase in immunoglobulin G (IgG) titre, detection of the non-structural protein 1 (NS1) and detection of dengue-specific immunoglobulin M (IgM) and IgG in a single serum sample (applicable before 2009). The annual numbers of suspected and laboratory-confirmed dengue cases were obtained from Taiwan CDC websites (available since 1998).^6^ The GBD estimates of annual dengue case count were obtained from the Global Health Data Exchange website.

Figure 1 shows that the number of laboratory-confirmed cases recorded by Taiwan CDC fluctuates greatly each year, with notable spikes of 15 732 and 43 784 in 2014 and 2015. In contrast, GBD estimates depict a trend of increasing cases, escalating from 341 187 in 1998 to a peak of 658 864 in 2017, and then slightly receding to 615 567 in 2019. These estimates are in marked disparity with official Taiwanese data, conspicuously missing the intensity of the 2014 and 2015 outbreaks. The discrepancy between GBD estimates and Taiwan CDC data ranges from 12- to 5240-fold.

**Figure 1:**
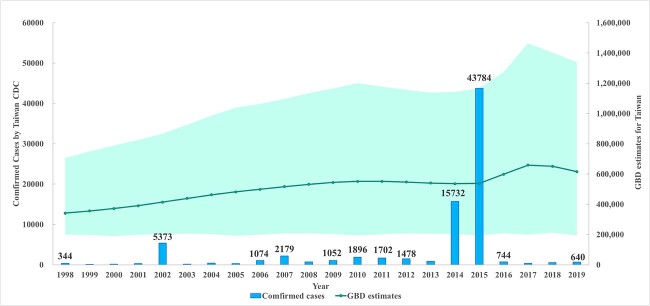
Annual counts of laboratory-confirmed dengue cases in Taiwan as reported by Taiwan CDC, compared with annual dengue case numbers in Taiwan estimated by the GBD, accompanied by 95% uncertainty intervals, Taiwan, 1998–2019

Taiwan, located on the Tropic of Cancer, hosts *Aedes aegypti*, an efficient transmission vector in the south, and *Aedes albopictus* island-wide. Despite these vectors, dengue has not become endemic in Taiwan, with outbreaks totally driven by imported cases, particularly where *A. aegypti* prevails.^7^ Fever screening at airports, initiated in 2003, aims to reduce case importation.^7^ Unlike Southeast Asia’s hyperendemic countries with all dengue serotypes and children mainly affected, Taiwan usually experiences outbreaks of a single serotype affecting adults. Between 2016 and 2022, most confirmed cases were imported, but a decline was noted post-2020 because of stringent coronavirus disease 2019 (COVID-19) quarantine policies and reduced international travel. Therefore, the annual variability of dengue cases in Taiwan, often peaking during infrequent severe epidemics, is not accurately captured by GBD estimates. We were unable to compare dengue death data between the Taiwan CDC and the GBD, as Taiwan CDC only provides death counts for selected years.^5^

Dengue cases are often underreported because of symptom similarity with other febrile illnesses and mild cases not seeking medical care. In Taiwan, before 2015, mandatory reporting of suspected cases by physicians, along with laboratory confirmation, was potentially hampered by delays in obtaining test results due limited availability of certified laboratories.^8^ This issue was exacerbated by the practice of pre-emptive insecticide spraying at patients’ homes, workplaces and schools, leading to complaints if subsequent tests proved negative. The widespread adoption of NS1 antigen rapid diagnostic tests in and post-2015 has markedly reduced underreporting, prompting physicians to quickly report and test suspected dengue cases to contain outbreaks swiftly. Every suspected case reported to Taiwan CDC undergoes testing, allowing the calculation of a positivity rate (Table 1)—an indicator broadly employed during the COVID-19 pandemic to assess transmission intensity.^9^ High positivity rates in 2014 and 2015 highlighted severe dengue epidemics in those years. In response, intensified vector control and surveillance by the local governments in southern Taiwan since 2016 have led to a notable decline in confirmed cases, evidenced by low positivity rates despite extensive testing. Therefore, it is recommended that governments should regularly disclose both confirmed case numbers and total tests administered. This practice facilitates a thorough analysis of surveillance efficacy and enables the determination of positivity rates. Consequently, this aids in assessing local transmission activity and distinguishing whether a rise in case numbers is because of increased transmission or intensified testing efforts.

**Table 1:** Annual numbers of reported, laboratory-confirmed dengue cases and test positivity rates for dengue virus infection amongst all cases, indigenous cases and imported cases in Taiwan, 1998–2022

	All			Locally acquired^d^			Imported^e^		
Year	Suspected^a^	Confirmed^b^	Positivity^c^	Suspected^a^	Confirmed^b^	Positivity^c^	Suspected^a^	Confirmed^b^	Positivity^c^
1998	1028	344	33.5%	984	309	31.4%	44	35	79.5%
1999	873	68	7.8%	832	42	5.0%	41	26	63.4%
2000	480	139	29.0%	447	113	25.3%	33	26	78.8%
2001	772	279	36.1%	706	227	32.2%	66	52	78.8%
2002	7590	5373	70.8%	7513	5321	70.8%	77	52	67.5%
2003	1060	145	13.7%	971	86	8.9%	89	59	66.3%
2004	1125	427	38.0%	993	336	33.8%	132	91	68.9%
2005	917	306	33.4%	743	202	27.2%	174	104	59.8%
2006	2179	1074	49.3%	2032	965	47.5%	147	109	74.1%
2007	3264	2179	66.8%	2995	2000	66.8%	269	179	66.5%
2008	1746	714	40.9%	1405	488	34.7%	341	226	66.3%
2009	1918	1052	54.8%	1609	848	52.7%	309	204	66.0%
2010	4241	1896	44.7%	3773	1592	42.2%	468	304	65.0%
2011	3936	1702	43.2%	3631	1545	42.6%	305	157	51.5%
2012	3602	1478	41.0%	3255	1271	39.0%	347	207	59.7%
2013	2714	860	31.7%	2265	596	26.3%	449	264	58.8%
2014	24 890	15 732	63.2%	24 500	15 492	63.2%	390	240	61.5%
2015	70 341	43 784	62.2%	69 791	43 419	62.2%	550	365	66.4%
2016	6881	744	10.8%	6098	381	6.2%	783	363	46.4%
2017	3500	343	9.8%	2674	10	0.4%	826	333	40.3%
2018	5620	533	9.5%	4578	183	4.0%	1042	350	33.6%
2019	9249	640	6.9%	7531	100	1.3%	1718	540	31.4%
2020	2371	137	5.8%	2140	73	3.4%	231	64	27.7%
2021	954	12	1.3%	904	0	0.0%	50	12	24.0%
2022	1823	88	4.8%	1755	20	1.1%	68	68	100.0%

^a^Suspected cases are those who have been reported to Taiwan CDC and received laboratory tests.

^b^Confirmed cases are suspected cases who meet criteria for laboratory confirmation of dengue virus infection.

^c^Positivity is the proportion of suspected cases that are laboratory confirmed.

^d^Cases are defined as ‘locally acquired’ if there is no travel history to a foreign country where dengue is endemic within 2 weeks before the onset of the illness.

^e^Cases are defined as ‘imported’ if there is a travel history to a foreign country where dengue is endemic within 2 weeks before the onset of the illness.

The GBD 2019 utilizes specific covariates, including the Health Care Access and Quality Index, the population-weighted probability of dengue infection, the cause-specific mortality rate and population density, for modelling dengue incidence.^2^ However, the complex epidemiology of dengue, characterized by intricate interactions amongst humans, vectors and the environment, renders this limited set of covariates insufficient for accurately capturing the disease's variability globally. In dengue non-endemic regions like Taiwan, the occurrence of outbreaks is more stochastic, making it even more challenging for the GBD's model that employs a smoothing approach to evaluate the yearly variation.^2^ Another notable limitation in the GBD's model is their reliance on 34 data points derived from 17 studies identified in their literature review to model the under-reporting adjustment factor for each country.^2^ However, this estimation may not fully account for the effects of the widespread adoption of rapid diagnostic tests in recent years, which could have notably reduced under-reporting.^2^ In fact, a serosurvey conducted at the end of the intense 2015 dengue epidemic in Tainan City, Taiwan, revealed minimal under-reporting, likely attributed to the widespread use of rapid NS1 tests and increased awareness from comprehensive public and medical campaigns in this severe epidemic.^10^

In summary, whilst the GBD study provides invaluable insights into global health trends, its estimates for dengue case numbers in Taiwan appear completely implausible. The GBD study's modelling approaches have limitations, particularly for diseases like dengue, where transmission dynamics vary within and between countries and where a mix of seasonal epidemics, periodic or multi-year interval epidemics can occur.

## Funding

This study was partially supported by National Cheng Kung University Hospital (NCKUH-11303004).

## Author contributions

Sin Yee Lee (Data curation [equal], Formal analysis [equal], Software [equal], Visualization [equal], Writing—original draft [equal]), Hsin-I Shih (Formal analysis [supporting], Investigation [supporting], Methodology [supporting], Writing—review & editing [supporting]), Chwan-Chuen King (Formal analysis [supporting], Investigation [supporting], Methodology [supporting], Writing—review & editing [supporting]), Tsung-Hsueh Lu (Conceptualization [equal], Methodology [equal], Resources [equal], Visualization [supporting], Writing—review & editing [supporting]) and Yu-Wen Chien (Conceptualization [lead], Data curation [equal], Formal analysis [lead], Funding acquisition [lead], Investigation [lead], Methodology [lead], Project administration [lead], Resources [lead], Supervision [lead], Validation [lead], Writing—original draft [lead])

##  

**Conflict of interest:** None declared.

## Ethics approval

This study was approved by the Institute of Review Board of National Cheng Kung University Hospital (A-EX-112-037).

## Editorial assistance

The authors would like to acknowledge the use of ChatGPT, an AI language model developed by OpenAI, for providing editorial assistance in improving the English language of this manuscript. It is important to note that the assistance provided by ChatGPT was limited to language editing, whereas all original content, ideas, data analysis, results and discussions were solely generated by the authors. After using this tool, the authors reviewed and edited the content as needed and take full responsibility for the content of the publication.

## Data availability

All data are publicly available at the Taiwan Centers for Disease Control website (https://nidss.cdc.gov.tw/) and the GBD 2019 database (https://vizhub.healthdata.org/gbd-results/).
